# Hypovitaminosis D is associated with negative outcome in dogs with protein losing enteropathy: a retrospective study of 43 cases

**DOI:** 10.1186/s12917-017-1022-7

**Published:** 2017-04-08

**Authors:** K. Allenspach, J. Rizzo, A. E. Jergens, Y. M. Chang

**Affiliations:** 1 Department of Clinical Sciences and Services, University of London, North Mymms, Hertfordshire UK; 2 Research Support Office, Royal Veterinary College, University of London, North Mymms, Hertfordshire UK; 3 Department of Clinical Sciences, College of Veterinary Medicine, Iowa State University, Ames, IA USA; 4 Department of Clinical Sciences, Iowa State University College of Veterinary Medicine, Ames, IA 50011 USA

**Keywords:** Protein losing enteropathy (PLE), Dog, Risk factors, Outcome, Vitamin D3, Ionized calcium

## Abstract

**Background:**

Hypovitaminosis D has previously been shown to be prevalent amongst dogs with protein losing enteropathy (PLE).

The hypothesis of this study was that Low 25-hydroxyvitamin D (25(OH) D) serum concentrations could be a risk factor for negative outcome in dogs with PLE.

Forty-three dogs diagnosed with PLE (2005–2014) and which serum Vitamin D serum concentrations were collected and archived at −80 Degrees C were analyzed.

Post-diagnostic communication with referring veterinarians was made to determine outcome of PLE dogss: Dogs which died due to PLE within 4 months after diagnosis (negative outcome group, *n* = 22) and dogs alive or which died due to another disease at the end point of the study (1 year after diagnosis, good outcome group, *n* = 21). Serum samples taken at the time of diagnosis were analysed for ionized calcium (iCa) concentrations and serum 25(OH) D concentration.

**Results:**

Clinical (CCECAI) scores, age at PLE diagnosis, and iCa concentrations were not significantly different between dog groups. A significantly greater (*p* < 0.001) number of PLE dogs treated with hydrolyzed or elimination diet alone showed good outcome as compared to the PLE negative outcome group. Median serum 25(OH) D concentration was significantly (*p* = 0.017) lower in dogs with negative outcome versus PLE dogs with good outcome. Using logistic regression analysis, 25(OH) D serum concentration was shown to be a statistically significant factor for outcome determination. Cox regression analysis yielded a hazard ratio of 0.974 (95% CI 0.949, 0.999) per each one nmol/l increase in serum 25(OH) D concentration.

**Conclusions:**

Low serum 25(OH) D concentration in PLE dogs was significantly associated with poor outcome. Further studies are required to investigate the clinical efficacy of Vitamin D (cholecalciferol) as a potential therapeutic agent for dogs with PLE.

## Background

Protein-losing enteropathy (PLE) in dogs is a clinical syndrome characterized by loss of protein through the intestines [1]. There are three major causes for PLE in dogs including inflammatory bowel disease (IBD), primary intestinal lymphangiectasia (IL), and intestinal lymphoma [1]. Apart from dogs diagnosed with intestinal lymphoma, which generally show poor response to chemotherapy and short survival times, dogs with PLE secondary to IBD or primary IL have a variable prognosis [1–5]. Only few reports describe prospective treatment trials of dogs with PLE since mortality is high despite intense immunosuppressive and nutritional treatment protocols [2, 3]. Possible life-threatening complications include intractable diarrhea, extreme malnutrition, and thromboembolic disease [6]. Risk factors associated with poor outcome have not been well characterized in PLE dogs to date. Several breeds are predisposed to the development of PLE, with Yorkshire Terriers having better outcome in some instances [4] while disease in Rottweilers generally carries a poor prognosis [1]. In addition, there is evidence that biomarkers, such as serum C-reactive protein, serum canine pancreatic lipase immunoreactivity, and fecal alpha-1 proteinase inhibitor concentrations, are more commonly elevated in those dogs having the shortest survival times [7, 8]. Electrolyte disturbances, such as low total and ionized calcium concentrations and hypomagnesemia, have also been reported in some PLE in dogs [9, 10]. It is hypothesised that the ionized hypocalcemia in IBD cases could be caused by reduced Vit D or calcium–absorption, reduced dietary intake, and/or Vitamin D receptor polymorphisms in impaired Vitamin D metabolism [11]. Furthermore, low serum concentrations of 25(OH) D have recently been described in dogs with chronic enteropathies [12], and have been shown to be associated with negative outcome [13]. We therefore sought to investigate the presence of low iCa and 25(OH) D serum concentrations in dogs with PLE and whether these variables were associated with negative outcome.

## Methods

### Aim, design and setting of the study

The aim of the current study was to assess the prevalence of decreased 25(OH) D serum concentrations in dogs with PLE caused by IBD. In addition, we investigated whether 25(OH) D could serve as a prognostic indicator of outcome.

This was a retrospective study including 43 cases seen at the Royal Veterinary College, University of London, over the time period of 2005–2014.

### Animals

The medical records of dogs referred to the Queen Mother Hospital for Animals (QMHA), Royal Veterinary College between 2005 and 2014 were reviewed retrospectively to identify dogs with a clinical diagnosis of PLE. The diagnosis of PLE was made if all of the following applied: (1) history of chronic gastrointestinal disease (including weight loss, vomiting, diarrhea, decreased appetite); (2) panhypoproteinemia (serum albumin less than 2.8 g/dL and serum globulin less than 2.1 g/dL; reference ranges 2.8–3.9 and 2.1–4.1 g/dL, respectively); (3) diagnostic tests including performance of complete blood count, biochemistry profile, urinalysis, abdominal ultrasound, ACTH stimulation test, serum trypsin like immunoreactivity (TLI), and canine pancreatic lipase immunoreactivity (cPLI) serum assays to reflect the presence or absence of primary GI disease versus extra-intestinal disease, (4) histopathological confirmation of IL or IBD with secondary IL; (5) exclusion of hepatic dysfunction by serum bile acid stimulation test; and (5) absence of proteinuria. Proteinuria was excluded in all dogs on the basis of a negative urine dipstick or a urine protein: creatinine ratio of <0.5. In addition, at the time of PLE diagnosis, all dogs had to have a clinical disease activity index (Canine Chronic Enteropathy Clinical Activity Index, CCECAI [14]) recorded by the clinician, and a serum sample frozen within 30 min after collection and stored at −80 Degrees Celsius until later analysis.

### Clinical data

Follow up communication with referring veterinarians was made to determine post-diagnostic outcome of PLE dogs. In accordance with previoulsy published studies, dogs were divided into two groups: The first group consisted of dogs which had died from their illness or were euthanized due to intractable clinical disease within 4 months after diagnosis [4] (negative outcome group), and the second group consisted of PLE dogs that were alive or had died due to non-PLE disease at least 1 year after diagnosis (good outcome group). Individual treatments of dogs were also categorized into two groups: Group 1 dogs comprised those who received either an elimination diet (single protein diet that the dog had not been given before, using a commercially available veterinary therapeutic diet) or a hydrolyzed diet (commercially available, hydrolyzed ingredient veterinary therapeutic diet) on an exclusive basis (diet group); Group 2 dogs consisted of dogs who were prescribed elimination or hydrolyzed diet in conjunction with immunosuppressive drugs, including combination therapy with prednisolone, cyclosporine, and/or azathioprine.

### Measurement of ionized calcium (iCa) and serum 25(OH) D concentrations

Vitamin D status was assessed by the measurement of serum concentrations of 25-hydroxyvitamin D (25[OH] D), which is the most widely used approach to analyze whole body vitamin D status [15]. At the time of diagnosis, dogs had samples collected for biochemical and hematological analysis. Residual serum samples were then frozen at −80 °C within 30 min after collection, until future analysis. Ionized calcium concentrations were measured using an ion specific electrode and 25(OH) D was measured using commercially available radioimmunoassays (RIA) that have been validated for use in veterinary medicine [16]. Samples were shipped on dry ice to the Michigan State University’s Diagnostic Center for Population and Animal Health, (DACPAH)^1^ for batch analysis. Serum 25(OH) D and iCa concentrations have previously been shown to be stable under these conditions [17], and DACPAH^1^, personal communication).

### Statistical analysis

Differences between dog groups were assessed using a Mann-Whitney *U*-test for numerical data or Fisher’s exact test for categorical data, respectively. Correlations were analysed using Spearman Rank correlation tests. Breed, age, serum albumin concentrations, CCECAI scores, treatment group, iCa concentrations and 25(OH) D concentrations were entered into a univariate logistic regression analysis. Factors that were significantly associated with outcome in the univariate logistic regression analysis were then assessed in a multivariable logistic regression. Kaplan-Meier curve and Cox regression analyses were used to illustrate and estimate the effect of 25(OH) D serum concentration on survival times after diagnosis. Hazard ratio (HR) and 95% confidence interval (CI) were reported. Statistical analyses were performed with SPSS version 22 and GraphPad Prism 7 statistical software, with a *p* < 0.05 considered statistically significant.

## Results

Forty-three PLE dogs were included in the study with 21 dogs having good outcome and 22 dogs having negative outcome. In the negative outcome group, median survival time was 19 days (range 1–301 days). In the good outcome group, 13/22 dogs were still alive at 4 months, while 9 dogs had been euthanized due to non-PLE related illnesss. Median survival time in this latter group was 1095 days (range 515–3130 days).

In the good outcome group, median age was 5.2 years (range 1–11 years), with six neutered males, three entire males, nine neutered females, and three entire females making up this group. Breeds in this cohort included 5 crossbreed dogs, 2 each of Miniature Schnauzers, Labrador Retrievers and Border Collies, and one each of American Bulldog, Weimaraner, Cavalier King Charles Spaniel, Cocker Spaniel, Griffon, Boxer, English Springer Spaniel, Jack Russel Terrier, Tibetan Terrier and Standard Poodle. Histopathological diagnoses in this group were IBD in 16 dogs, and IBD with IL in five dogs. Median age in the negative outcome group was 6.7 years (range 0.9–13.7 years), with four neutered males, four entire males, twelve neutered females, and two entire females in this group. Breeds included in the negative outcome cohort included three dogs each of Cavalier King Charles Spaniel and Golden Retrievers, two each of Cocker Spaniels and Dogue de Bordeaux, and one each of Greyhound, Schnauzer, Toy Poodle, Border Terrier, Kerry Blue Terrier, Rottweiler, Shar Pei, Weimaraner, Boxer, Staffordshire Terrier, Yorkshire Terrier, and Crossbreed dog. Histopathology in this group was consistent with IBD in 13 dogs, IBD with IL in four dogs, and IL only present in five dogs. There was no statistically significant difference in age or breed distribution between the two PLE dog groups (*p* = 0.35 and *p* = 0.42, respectively). Median Body Condition Score (BCS)^2^ was not different between the two groups (group with good outcome 4.5 (range 1–6), and group with negative outcome 3.8 (range 1–5), *p* = 0.5).

The percentage of dogs receiving immunosuppressive drugs between outcome groups was significantly different, with the negative outcome drugs receiving more immunosuppressive drugs (*p* < 0.001). A greater number of dogs treated with diet alone were in the good outcome (13/22) group versus PLE dogs in the negative outcome group (2/21, *p* < 0.001).

Median serum albumin concentration was 17 g/l (reference range 28–35), with no difference observed between the outcome groups (good outcome group: median 19, range 12–26; negative outcome group: median 16, range 10–27, *p* = 0.23). Serum albumin concentration was not correlated with either iCa, 25(OH) D or CCECAI (r^2^ = 1.15; r^2^ = 0.21; and r^2^ = 0.004, respectively).

**Fig. 1:**
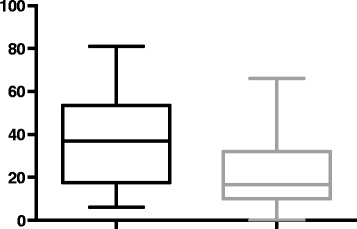
*Box* and *Whisker plots* representing 25(OH) D serum concentrations between PLE dogs in the poor outcome versus good outcome groups. (25(OH) D serum concentration in poor outcome group: median 16.5 nmol/L, range 0–66 nmol/L; good outcome group: median 37 nmol/L, range 6–81 nmol/L, *p* = 0.017)

**Fig. 2:**
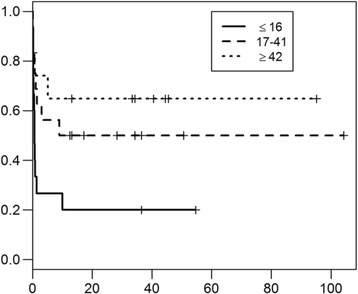
Kaplan-Meier curve and Cox regression using >16 nmol/l, 117–41 nmol/l, and >42 nmol/l as cut-off points for 25(OH) D serum concentration. Higher (25(OH) D serum concentration at diagnosis indicated a better survival of PLE dogs with an hazard ratio of 0.974 (95% CI 0.949, 0.999) per one nmol/l increase in vitamin D

The median 25(OH) D concentration was 23 nmol/L (range 0–81 nmol/L, [reference range 60–215 nmol]), being significantly lower in the negative outcome group (16.5 nmol/L, range 0–66 nmol/L) versus the good outcome group (37 nmol/L, range 6–81 nmol/L, *p* = 0.017) (Figure 1). Hypovitaminosis D was present in 17 dogs (81%) of the good outcome group and was not statistically different (*p* = 0.65) than its occurrence in the 20 dogs (91%) of the negative outcome group [reference range 60–215 nmol]). Higher 25(OH) D serum concentration at PLE diagnosis indicated better prognosis for survival with a hazard ratio of 0.974 (95% CI 0.949, 0.999) for each one nmol/l increase in 25(OH) D serum concentration (Figure 2).

Serum iCa concentrations were measured at the time of diagnosis in 41 of the 43 patients. The median serum iCa concentration in the combined cohorts of PLE dogs was 1.22 mmol/L (range 0.79–1.45 mmol/L, [reference range 1.25–1.45 mmol/L]). In the good outcome group (*n* = 21), the median serum iCa concentration was 1.25 mmol/L (range 0.79–1.35 mmol/L) with 10 dogs having iCa concentration below reference range. In the negative outcome group (*n* = 20), the median serum iCa concentration was 1.18 nmol/L (range 0.84–1.45 mmol/L), with 13 dogs having iCa concentrations below the reference range. There was a moderate positive correlation between serum iCa and 25(OH) D concentrations (*r* = 0.52, *p* < 0.0005).

The CCECAI scores between the good versus negative outcome groups were not statistically significant (negative outcome group: median 8, range 4–19; good outcome group: median 7, range 4–13; *p* = 0.6). There was no correlation between CCECAI scores or BCS and 25(OH) D concentrations (CCECAI: *r* = 0.043, *p* = 0.786; BCS: *r* = 0.069, *p* = 0.465). Treatment with immunosuppressive drugs and low serum 25(OH) D concentration at diagnosis were the only factors associated with negative outcome (univariate logistic regression: *p* = 0.006 and *p* = 0.024, respectively). 25(OH) D serum concentration was the only significant (*p* = 0.033) risk factor in the multivariable logistic regression analysis, with an increase of 25(OH) D level reducing the odds of having a poor outcome (odds ratio = 0.96, 95% confidence interval: 0.93–0.997).

## Discussion

Decreased iCa serum concentrations have previously been described with PLE possibly due to malabsorption of Vitamin D in dogs with severe mucosal disease [10]. This study shows for the first time that low 25(OH) D serum concentrations and low iCa serum concentrations are highly prevalent in a cohort of PLE dogs, and that decreased 25(OH) D serum concentrations are significantly associated with negative outcome.

There was no significant correlation between age and outcome of PLE patients, similar to an earlier report [10]. We also could not confirm any breed associations with poor outcome; however, this lack of association was likely influenced by the low number of susceptible breeds (e.g., Rotties and Yorkies) found in the study population.

There was a significant correlation between treatment group (diet versus diet + drugs) and outcome of PLE patients. The majority of patients in the good outcome group were managed solely with nutritional therapy, while the majority of patients in the poor outcome group were treated with diet and immunosuppressive drug protocols. Clinical disease severity, as determined by CCECAI and BCS at the time of diagnosis, was not significantly different between the two groups suggesting that disease activity was not a significant variable affecting outcome. The fact that BCS was not different between the PLE groups also indicates that poor nutritional status alone was not predictive of outcome. Finally, there was no significant correlation between CCECAI scores, BCS, and 25(OH) D concentration at diagnosis with regards to outcome prognosis. It is therefore likely that the treatment group was merely a marker for response to management, and therefore not an independent predictor for outcome. Moreover, the BCS system used in this system has not been independently validated and it is therefore possible that more sensitive methods to assess body condition, such as bone density measurement (DEXA), may have shown different results.

Serum albumin concentration was not identified as a negative prognostic indicator in this cohort of dogs with PLE. In previous publications, albumin was found to be correlated with poor prognosis in dogs with chronic enteropathies in general [14, 16] as well as in Yorkshire Terriers with PLE [4]. However, other studies investigating canine PLE were unable to correlate albumin with negative outcome [7, 13]. It is possible that serum albumin is more important as a prognostic indicator when it is only slightly to moderately below the reference range [14] and less important once the albumin concentration is severely low. In addition, we could not find a correlation between serum albumin concentration and iCa, serum 25(OH) D concentrations or CCECAI. This indicates that loss of Vitamin D-binding protein alone is probably not the sole factor for decreased serum 25(OH) D concentrations in these dogs. Furthermore, it may indicate that serum 25(OH) D concentration is an important metabolite to measure in these patients, as serum albumin alone may not be predictive for outcome.

Several studies have described dogs with gastrointestinal disease and low total and iCa serum concentrations are often prone to hypocalcemia even after clinical improvement [9, 18, 19]. This could possibly be due to serum vitamin D levels not being corrected and/or increased fraction of serum ionized calcium. In humans with vitamin D deficiency, survival is significantly better in patients with normal vitamin D levels compared to severely ill patients with vitamin D deficiency [20]. In addition, median serum concentrations in the group with poor outcome were in the deficiency range for people as defined by the Institue of Medicine Consensus Guidelines,^3^ whereas those dogs in the group with good outcome had median 25(OH) D serum concentrations in the insufficient range. There are no official guidelines available for dogs, but the data presented here suggest that clinicians should consider measuring 25(OH) D serum concentrations in dogs with PLE and possibly supplement this vitamin in deficient cases.

A limitation of this study is its retrospective nature and therefore, the interpretation of clinical data used in this study. In addition to the fact that the non- standardized treatment approach could have biased the study, it is also possible that other confounding variables, such as previous treatment protocols and dietary intake before diagnosis were not accounted for. The use of RIAs has historically been less accurate than the gold standard techniques of liquid chromatography-mass spectometry (LC-MS). Furthermore, RIAs recognize both 25(OH) D as well as 24, 25(OH) D_2_ and other polar metabolites, and therefore may overestimate 25(OH) D levels by approximately 10–20% as compared to LC-MS. This fact should be taken into account when interpreting the results of this study. The RIA used in this study has an intra-assay repeatability (12 replicates), % coefficients of variation for serum pools of 30, 109, and 183 nmol/L of 5.0, 4.6, and 4.2%, respectively. For interassay repeatability (13 assays), the % coefficients of variation serum pools of 32 and 115 nmol/L are 15.3 and 10.3%, respectively [16].

Future studies investigating vitamin D status in dogs should be performed using the gold standard tests as well as standard quality control schemes for laboratories, such as the Vitamin D External Quality Assurance Scheme (DEQAS^4^).

It is important to note that an association between low 25-hydroxyvitamin D and outcome in this cohort of PLE dogs was found, however, this does not imply any causal association between the two parameters.

## Conclusions

This is the first study to demonstrate a link between low vitamin D serum concentration and outcome in canine PLE patients. Further studies are required to investigate calcitriol as a potential adjuvant therapeutic agent in PLE patients as has been shown in other models.

## Abbreviations

25(OH) D: 25-hydroxyvitamin D
BCS: Body Condition Score
CCECAI: Canine Chronic Enteropathy Clinical Activity Index
DEQAS: Vitamin D External Quality Assurance Scheme
HPLC: High-performance liquid chromatography
IBD: Inflammatory bowel disease
IL: Intestinal lymphangiectasia
LC-MS: Liquid chromatography-mass spectrometry
PLE: Protein losing enteropathy
QMHA: Queen Mother Hospital for Animals

## Footnotes

1 MSU Diagnostic Center for Population and Animal Health, Meridian Charter Township, MI, USA

2 WSAVA Body Condition Score for Dogs http://www.wsava.org/sites/default/files/Body%20condition%20score%20chart%20dogs.pdf

3
http://www.nos.org.uk/document.doc?id=1352

4
http://www.deqas.org
